# Dietary flavanols improve cerebral cortical oxygenation and cognition in healthy adults

**DOI:** 10.1038/s41598-020-76160-9

**Published:** 2020-11-24

**Authors:** Gabriele Gratton, Samuel R. Weaver, Claire V. Burley, Kathy A. Low, Edward L. Maclin, Paul W. Johns, Quang S. Pham, Samuel J. E. Lucas, Monica Fabiani, Catarina Rendeiro

**Affiliations:** 1 Beckman Institute for Advanced Science and Technology, University of Illinois at Urbana-Champaign, Urbana, IL USA; 2 Department of Psychology, University of Illinois at Urbana-Champaign, Urbana, IL USA; 3 School of Sport, Exercise and Rehabilitation Sciences, University of Birmingham, Birmingham, B15 2TT UK; 4 Abbott-Nutrition Division, Research and Development, 3300 Stelzer Road, Columbus, OH 43219 USA; 5 Centre for Human Brain Health, University of Birmingham, Birmingham, UK

**Keywords:** Neuroscience, Physiology, Psychology

## Abstract

Cocoa flavanols protect humans against vascular disease, as evidenced by improvements in peripheral endothelial function, likely through nitric oxide signalling. Emerging evidence also suggests that flavanol-rich diets protect against cognitive aging, but mechanisms remain elusive. In a randomized double-blind within-subject acute study in healthy young adults, we link these two lines of research by showing, for the first time, that flavanol intake leads to *faster* and *greater brain* oxygenation responses to hypercapnia, as well as *higher performance* only when cognitive demand is high. Individual difference analyses further show that participants who benefit from flavanols intake during hypercapnia are also those who do so in the cognitive challenge. These data support the hypothesis that similar vascular mechanisms underlie both the peripheral and cerebral effects of flavanols. They further show the importance of studies combining physiological and graded cognitive challenges in young adults to investigate the actions of dietary flavanols on brain function.

## Introduction

Lifespan wear and tear of the vascular system due to poor nutrition and lack of fitness, among other factors, can accelerate cognitive aging and lead to dementia. There is epidemiological evidence suggesting that flavonoids, a group of small molecules present in fruits and vegetables, can protect against vascular disease and cardiovascular-related mortality^1–4^. In particular, cocoa flavanols, a sub-group of flavonoids (also present in berries, grapes, apples and tea) have been shown to improve endothelial function in humans quite rapidly (within 1–2 h) by enhancing vasodilatory properties of peripheral arteries^5,6^. Acute benefits translate effectively into short-term (2–8 weeks) clinically relevant improvements in blood pressure and endothelial function (as measured by brachial flow-mediated dilatation, FMD)^7,8^, comparable to those of drugs, such as statins^9,10^. Mechanistically, the beneficial effects of cocoa flavanols on endothelial function have been linked to increases in bioavailability of nitric oxide (NO)^6^, which is known to be affected in the earliest stages of vascular disease^11^.Whilst the acute effects of flavanols have been mainly attributed to phase I/II-derived (−)-epicatechin metabolites, the short to long term benefits may be also driven by gut-derived metabolites^12,13^, although this remains to be established. Another emerging line of research further suggests that this class of plant-derived compounds may protect against cognitive decline in aging^14–16^ and cognitive resilience to neuropsychiatric disorders and stress^17,18^. Yet, the extent to which increases in circulatory levels of NO by flavanols can translate into benefits in the brain vasculature, and effectively influence cognitive performance in humans, is poorly understood.

Cerebral blood flow is controlled by neuronal activity but also by levels of arterial blood gases, in particular carbon dioxide (CO_2_)^19^. Relevant to our hypothesis is the fact that NO is known to contribute to CO_2_-dependent increases in cerebral blood flow in humans (hypercapnia)^20^. Furthermore, cerebrovascular reactivity to CO_2_ is widely accepted as a key biomarker of cerebrovascular health, and has been closely associated with cognitive function in health and disease states^21–25^. Hence, hypercapnia represents a robust model to test whether flavanol-mediated increases in endothelial function (as assessed by gold-standard FMD) mediate benefits in cerebrovascular and cognitive function.

Only a handful of studies have previously reported effects of flavanols on the human cerebral vasculature, both in a resting state^26–29^ and in response to cognitive challenges^30–32^, albeit in opposite directions (increase/decrease in blood flow/velocity). Further, modulation of cerebral physiological outcomes by flavanols in the context of neuronal/cognitive challenges frequently and surprisingly fail to translate into cognitive benefits^26,30–32^. A possibility is that the benefits of flavanols may only be visible at high levels of task difficulty. This highlights that, whilst some of these studies could provide ecological validity (as they target aging adults with cognitive and/or vascular problems), they were not designed in a manner that allows for an evaluation of the underlying physiological effects of these compounds in the human brain. This leaves some uncertainty about whether flavanols’ benefits in peripheral vascular function are reflected by similar effects on cerebrovascular reactivity, and whether the cognitive and vascular benefits are related.

There is a need to determine whether the peripheral vascular benefits of flavanols extend to the cerebral vasculature. This requires well-controlled experiments to demonstrate that flavanols (a) can modulate the brain’s vasculature; (b) that these effects, similarly to those found in the periphery, are revealed during physiological challenges likely involving the NO pathway; (c) that they affect cognitive performance, at least in challenging conditions; and (d) that cerebrovascular and cognitive benefits are linked.

In the current study, we employed separate physiological and cognitive challenges in a double-blind, within-subject, placebo-controlled acute (2 h) study to assess the underlying physiological actions of cocoa flavanols on cerebral and peripheral vascular and cognitive function. To measure cerebrovascular reactivity, we employed a CO_2_-breathing challenge (hypercapnia) before and after intake of either a high- or low-flavanol intervention. During hypercapnia, we measured cortical haemoglobin concentration using functional near-infrared spectroscopy (fNIRS)^33^, which allowed us to finely quantify the dynamics of cerebrovascular reactivity, providing information not only about the amplitude of this response, but also about its time course. Similar to peripheral measures, cerebral CO_2_-reactivity has been shown to be mediated by the NO pathway^20^, which is the hypothesized mechanism underlying the beneficial effects of flavanols on peripheral endothelial function^6^. To measure the cognitive effects of flavanols, we employed tasks with escalating levels of difficulty^34^, which could inform us of the level at which the cognitive benefits of flavanols may emerge.

## Results

### Dietary flavanols improve cerebral oxygenation responses to hypercapnia

**Figure 1:**
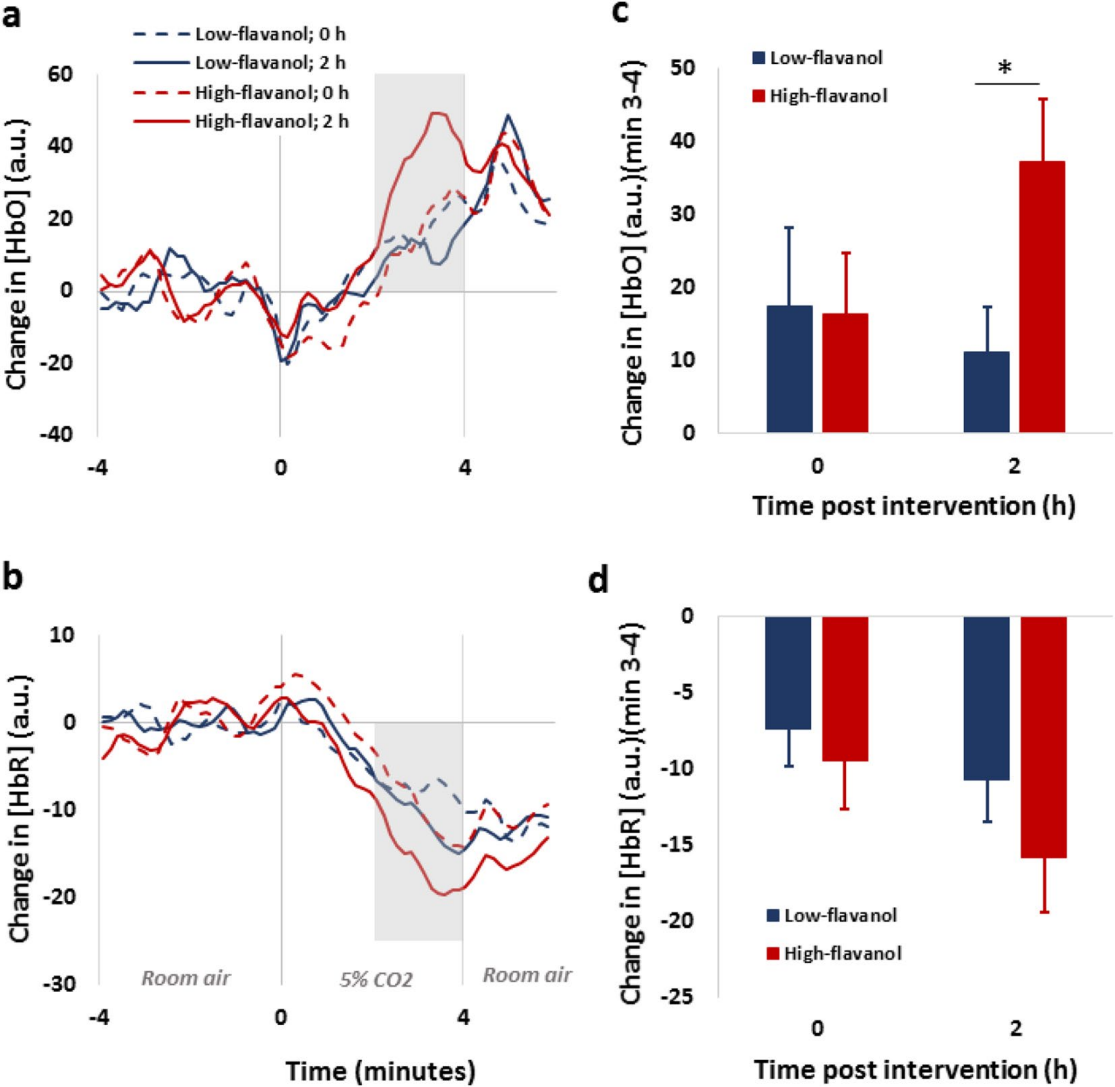
Haemodynamic responses in frontal cortical regions during hypercapnia (5% CO_2_) both before (0 h) and after (2 h) intake of either a low- or high-flavanol dietary intervention. Time course for Oxygenated (**a**) and Deoxygenated-haemoglobin (**b**) are presented as relative changes from baseline (room air) and averaged across participants (N = 17) and across frontal brain locations. The average relative concentrations of oxy- (**c**) and deoxy haemoglobin (**d**) during minutes 3–4 of the CO_2_ challenge (corresponding to maximal dilation) show that the high-flavanol intervention induces significantly higher oxygenation levels than the low-flavanol at 2 h post-intervention (*p = 0.030). No significant differences are observed for deoxygenated haemoglobin (Mean ± SEM).

Figure 1 reports the time course of oxy-(Fig. 1a) and deoxy-haemoglobin (Fig. 1b) concentration, averaged across cortical recording locations and participants, during the 5% CO_2_-breathing challenge (Fig. S1 shows mean ± SEM for both the oxygenation and deoxygenation time courses). Separate time courses are shown for the high- and low-flavanol conditions and for the measurement points before and following the flavanol intervention, where increases in oxy-haemoglobin and reductions in deoxy-haemoglobin concentration are associated with vasodilation induced by the hypercapnia. Two-way repeated-measure ANOVAs (with dietary intervention and time of measurement as factors) indicated that high-flavanol intake was associated with a larger oxygenation response: the interaction was significant for the amplitude of the response, measured as the average change during the period 3–4 min after CO_2_-breathing onset, ***F***(1,16) = 6.08, *p* = 0.025 (Fig. 1c). Simple-main effects analyses revealed no differences at baseline (*p* = 0.993), but a significantly greater oxy-haemoglobin level after the high-flavanol in comparison to the low-flavanol (*t*(16) = − 2.37, *p* = 0.030) (1c). No significant differences between interventions were detected for deoxygenated haemoglobin (Fig. 1d).

**Figure 2:**
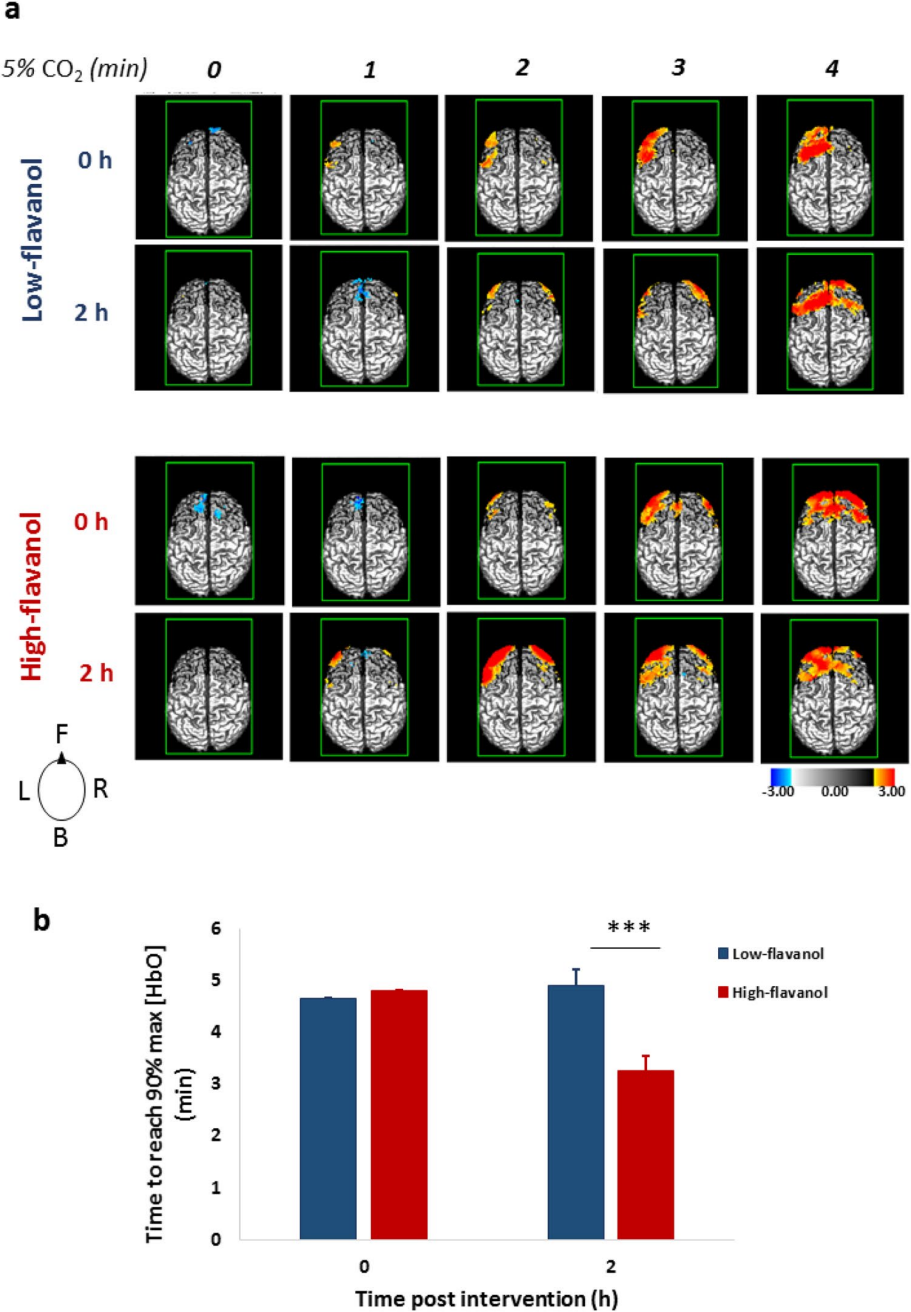
Brain maps of oxygenated haemoglobin during hypercapnia (5% CO_2_) both before (0 h) and after (2 h) intake of either a low- or high-flavanol dietary intervention. (**a**) Oxy-haemoglobin concentration changes (in *z* scores) across frontal cortical regions and averaged across participants (N = 17) are presented at the time of onset (0 h) and 1, 2, 3, and 4 min into the CO_2_ challenge. Brain maps, viewed from the top, are oriented as indicated in the inset diagram: F = front, B = back, L = left, R = right; Darker grey background color indicates the recording area. (**b**) Latency to reach 90% maximal blood oxygenation was significantly lower (***p < 0.001) by approximately 1 min after the high-flavanol intervention in comparison to low-flavanol (Mean ± SEM).

Figure 2 reports maps of the oxy-haemoglobin concentration changes (in *z* scores) at different times during the CO_2_-breathing challenge (at onset, and 1, 2, 3, and 4 min into the challenge), with darker grey background indicating the recording region. The maps show that increases in blood oxygenation were most evident in lateral frontal regions and that high-flavanol intake led to earlier and larger responses than the other three conditions, which were similar to each other (Fig. 2a). There was a significant interaction between intervention and the latency of response, measured as the time to reach 90% maximal oxygenation, *F*(1, 16) = 13.61, *p* = 0.002 (Fig. 2b), based on a jack-knife approach^35^. There were no significant differences at baseline (*p* = 0.429), but latency of oxygenation was significantly shorter (by approximately 1 min) after the high-flavanol intervention (*t*(16) =  − 5.68, *p* < 0.001).

Efficacy of the high-flavanol intervention on endothelial function, as measured by brachial FMD (Fig. S2), was confirmed, with significant increase of approximately 1% FMD after the high-flavanol in comparison to the low-flavanol intervention (*p* =  < 0.001), as previously described in this population^8^.

### Dietary flavanols improve cognitive performance when cognitive demand is high

**Figure 3:**
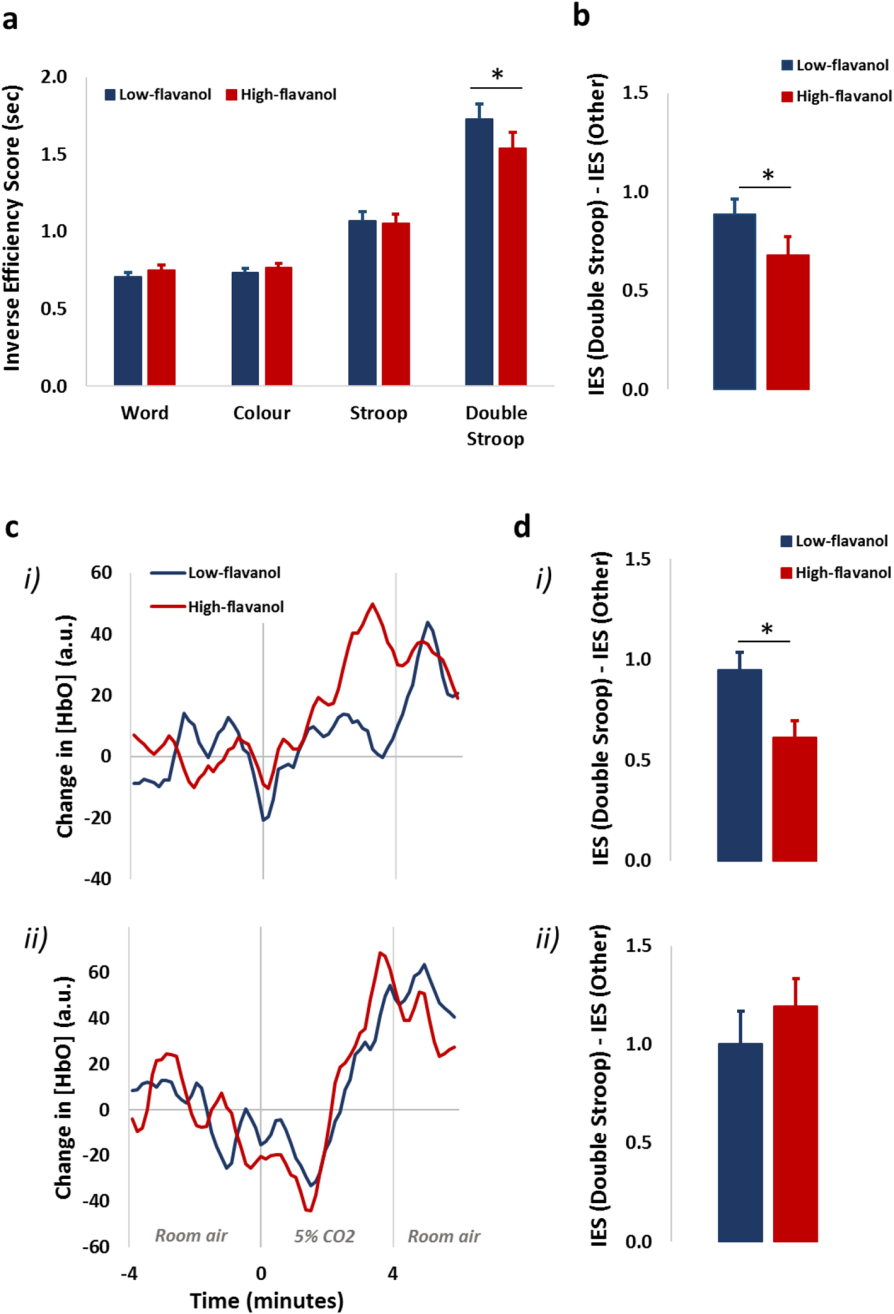
Cognitive performance in a modified version of the Stroop task 2 h after intake of either a low- or high-flavanol dietary intervention. Inverse efficiency score (IES) is described as reaction time/accuracy (expressed in seconds) during cognitive performance across four different conditions representing progressively harder cognitive demands (**a**). The Double-Stroop condition induces the slowest cognitive processing (higher IES) compared to the other conditions (word, colour and Stroop). The benefit of the high-flavanol intervention (lower IES) in only apparent in the Double Stroop condition, when the level of conflict is higher. The cognitive benefit of high-flavanol cocoa in the Double Stroop over and above the other cognitive tasks is significantly higher in comparison to the low-flavanol intervention (*p = 0.045) (**b**). Two sub-groups of volunteers, (i): N = 13 and (ii): N = 4, revealed distinct oxy-haemoglobin benefits from flavanols during hypercapnia (**c**); with only the sub-group (i) showing benefits from flavanols during both the hypercapnia challenge and the cognitive task (**d**). The data is presented as Mean ± SEM.

Figure 3 reports the behavioural effects of flavanol intake, using the inverse efficiency score (IES), a parameter summarizing reaction time and accuracy during behavioural tasks^36–38^. A lower IES value (expressed in seconds) indicates more efficient cognitive processing. As shown in Fig. 3a, the four different conditions represented progressively harder cognitive challenges. The Double-Stroop condition, in which conflict was present both for stimulus classification and response selection for maximum difficulty (Fig. S4), induced the slowest responses.

Results indicate that only the Double-Stroop task differentiated between the high- and low-flavanols conditions. The interaction in the two-way repeated-measure ANOVA (with task condition and flavanol intervention as factors, and flavanol intervention order used as a covariate) was significant, *F*(3,48) = 3.267, *p* = 0.029. A planned contrast between the difference in the Double-Stroop and the average of the other three conditions yielded a significant cognitive advantage in the high flavanol *versus* low-flavanol condition, *t*(16) = 2.142, *p* = 0.042 (3b). The contrast of the Stroop task with the other two tasks was not significant, *t*(16) = 1.050, *p* = 0.309, confirming that flavanols only generated an advantage in the most difficult condition (Double-Stroop), but not in the other conditions.

### Cerebrovascular reactivity effects predict behavioural effects across individuals

In the majority of individuals (13 out of 17, see Fig. 3c (i)) the haemodynamic oxygenation responses to hypercapnia matched the overall average and showed a faster and larger cerebrovascular response to the CO_2_-breathing challenge after the intake of high-flavanol in comparison to the low-flavanol intervention. However, some individuals (4 out of 17, see Fig. 3c (ii)) did not follow this pattern. To understand this better, we computed separate average time courses of the oxy-haemoglobin concentration changes induced by hypercapnia under high- and low-flavanols for these two groups (Fig. 3c). This figure indicates that, for the latter group (ii), the fNIRS waveforms were very similar after high- and low-flavanols intake (*r* = 0.864, indicating a strong replicability of the fNIRS waveforms across sessions), and reached a maximum lagged cross-correlation (*r* = 0.897) when the high-flavanols waveform was anticipated by 18 s relative to the low-flavanols waveform. In contrast, in most participants (N = 13), the fNIRS waveforms were not as strongly correlated at lag 0 (*r* = 0.567), but reached a much higher cross-correlation (*r* = 0.807, again suggesting good reproducibility) when the high-flavanols waveform was anticipated by 117 s relative to the low-flavanol waveform (i), the phenomenon reflected by the full group analyses (Fig. 1).

Crucially, cerebrovascular reactivity to flavanols for these two sub-groups was predictive of the responsiveness to flavanols under the cognitive challenge. Figure 3d shows the behavioural interaction term (IES in the Double Stroop minus IES in other task conditions after high-flavanol relative to low-flavanols intake) in the two subgroups. The majority of participants (group *i*., i.e., individuals showing flavanols-induced improvements in cerebrovascular reactivity to CO_2_) showed a clear and significant improvement in performance in the Double Stroop (*t*(12) = − 3.784, *p* = 0.003, N = 13), whereas group *ii* (i.e., those individuals not showing flavanols-induced improvements in cerebrovascular reactivity to CO_2_) did not (*t*(3) = 0.86, *p* = 0.453, N = 4). This highlights the coupling of these two effects: those individuals showing improved cerebrovascular reactivity after high-flavanol intake also showed benefits during cognitive performance, whilst those that did not improve cognitive performance had no increase in cerebrovascular reactivity following high-flavanol intake.

## Discussion

The present study shows, for the first time, that cocoa flavanols lead to more efficient tissue oxygenation responses in frontal areas of the brain during a CO_2_-challenge in healthy young individuals. This suggests that, similarly to peripheral vascular benefits, flavanols result in clinically relevant improvements in cerebrovascular reactivity in a healthy brain. Such benefits further translate into improvements on prefrontal-dependent cognitive performance, but only for the highest level of cognitive demand. More importantly, only individuals who benefited from flavanol intake during hypercapnia experienced cognitive benefits, suggesting that these effects are likely linked. Finally, we confirmed that flavanol’s benefits on peripheral endothelial function (1% brachial FMD) are in line with those reported in previous studies^7,39^ in this population within an acute timeframe (2 h), where maximal levels of flavanol metabolites reach circulation in humans^13^.

### Flavanols improve cerebrovascular reactivity in young adults

The use of fNIRS allowed us for the first time to observe how flavanol intake can modify the dynamics of cerebral tissue oxygenation: our data suggest that improved efficiency of the coupling between hypercapnia and vasodilation is driven by not only larger but also faster tissue oxygenation (Figs. 1, 2). This clearly indicates rapid acute improvements in cerebrovascular reactivity in the frontal cortex and is consistent with acute improvements in peripheral endothelial function in this population shown previously^8^ and replicated in this study (approx. 1% FMD improvement; *see* Supplementary Material Fig. S2). Flavanols’ ability to increase bioavailability of circulating NO in humans is believed to underlie its benefits on peripheral endothelial function^6,40^ and we suggest that this mechanism is also contributing to the benefits observed in cerebrovascular reactivity. Although a direct causal link between NO and hypercapnia-induced vasodilation in humans remains to be established^41,42^, previous studies have shown that inhibition of NO results in decreased reactivity to hypercapnia in cerebral arteries^43,44^. More importantly, pharmacological interventions that directly increase NO production and improve endothelial function, such as NO-donor sodium nitroprusside^20,45^ and L-Arginine (substrate for endothelial NO synthase)^46^ also result in improvements in CO_2_ reactivity of cerebral vessels in humans. This is consistent with the hypothesis that flavanols enhance hypercapnia-induced release of NO from endothelial cells locally within the cerebral vasculature.

Recent evidence further suggests that hypercapnia induces vasodilation in the internal carotid artery (ICA, which supplies the frontal cortical areas assessed in the present study) by inducing increases in shear-rate^47,48^, which normally stimulates the production of NO by the endothelium^49^. This suggests a similar mechanism to brachial FMD, in which increases in shear-rate following hyperaemia result in NO-dependent arterial dilation^50,51^. As such, more efficient tissue oxygenation after flavanol intake might also be the result of NO-mediated increases in dilation of upstream cerebral conduit arteries (e.g., ICA) in response to hypercapnia.

Previous studies that assessed the impact of flavanols on the cerebrovasculature employed other imaging methods (fMRI, Transcranial Doppler) either at rest^27,28^ or during cognitive manipulations^30,31^, producing contradictory results (increases and declines in blood flow/velocity). However, neither of these contexts engages directly with NO within the vasculature, which might contribute to the discrepancy in outcomes.

In physiological, real-world conditions, flavanol-induced faster vascular reactivity may help recovery from injuries such as mild brain injury or stroke, conditions that are also associated with impaired cerebrovascular reactivity to CO_2_^52–54^. Cerebrovascular reactivity to CO_2_ is also depressed in populations at higher risk for cardiovascular disease and cerebral small-vessel disease, as well as in aging^45,55,56^. Importantly, lower cerebrovascular reactivity has been consistently associated with cognitive impairments^57^, and shown to be predictive of future cognitive decline^24^. Therefore, if the acute benefits demonstrated in the present study were to be sustained by continued intake of flavanol-rich foods (e.g., grapes, apples, cocoa, berries, tea), this may be particularly beneficial for populations at higher risk.

### Flavanols improve cognitive performance only when level of cognitive demand is high

Our behavioural findings indicate that flavanols can improve cognitive performance acutely in healthy young adults, but only when the level of cognitive demand is high (i.e., Double Stroop task; Fig. 3a,b). This indicates that assessment of flavanols’ effects on cognition requires challenging situations. The few previous acute studies in healthy young individuals showed inconsistent benefits across a variety of tasks, and, in particular, no benefits on a standard Stroop task^31^, a finding we replicate in our study. It has been previously suggested that failure to find cognitive effects in healthy young participants might be related to a high level of cognitive ability in this population, which is unlikely to be improved upon^26^. We suggest that the level of cognitive and physiological demands associated with the task are key factors, and we clearly demonstrate this by varying the level of conflict in the cognitive task used. Most interestingly, this indicates that flavanols may help cognition only when there is a substantial demand for information processing at the neuronal level, which, in turn, requires appropriate levels of blood oxygenation. Given the benefits in efficiency of cortical oxygenation we observed in the present study, we propose that flavanols may facilitate increases in blood oxygenation during complex tasks as a direct consequence of increased information processing demands in frontal cortical areas of the brain. Diets rich in dietary flavanols might be particularly beneficial when executive function becomes more limited (e.g., in older adults or individuals at higher risk for cognitive decline). Future work should focus on systematically employing difficulty-graded cognitive challenges when assessing the efficacy of dietary flavonoids on human cognitive function.

### Cognitive and cerebrovascular individual responses to flavanols are linked

Although measured separately, the extent of flavanol’s effects on measures of cerebrovascular reactivity in different individuals were predictive of their effects on cognitive measures in the same individuals. Specifically, only individuals for whom flavanols were effective in improving cerebrovascular reactivity (Fig. 3c) also showed behavioural improvement in challenging cognitive conditions (Fig. 3d), indicating a direct link between the cerebrovascular and cognitive effects of flavanols. This is the first time that such a relationship between hemodynamic and cognitive benefits of flavanols has been shown in a young healthy sample after a single dose of flavanols. Only one long-term flavanol intervention (3 months) study in older individuals has previously shown this link, with hippocampal-cognitive benefits being paralleled by increases in blood volume within the hippocampus (fMRI)^29^. Prior studies failed to link directly neurovascular benefits with cognitive benefits, when these are assessed simultaneously. For example, benefits in cognitive performance failed to reflect parallel improvements in neurovascular coupling, whilst changes in neurovascular coupling after flavanols did not result in cognitive improvements^30,31^. The disconnection between physiological and cognitive outcomes may be confounded by individual differences in responses to cognitive tasks. It is also possible that in some of these studies the cognitive tasks were not sufficiently challenging to demonstrate this relationship, as our results suggest.

Demonstration of this relationship has one important implication: there are individual differences in the way in which young healthy adults respond to the intake of flavanols. In the current study, we observed that those individuals with negligible cerebrovascular benefits from flavanols (N = 4) were individuals with particularly large and rapid responses to hypercapnia at baseline (or after low-flavanol intake). This indicates that lack of flavanol efficacy may be due to a ceiling effect, with high baseline cerebrovascular reactivity leaving little room for improving it further. Most remarkable is the observation that these same individuals did not benefit cognitively from flavanol intake. In contrast, after flavanol intake, individuals with lower baseline cerebrovascular reactivity to hypercapnia were brought up to levels similar to those of the ‘high performers’, effectively equalizing individuals.

Variability of the effects of flavanols on human vascular function have been reported previously and partially attributed to variations in flavanol metabolism and/or absorption: previous research reported up to 39% variability in flavanol plasma concentration after acute intake of flavanols in young adults^58^. However, previous research also suggests that these factors alone cannot explain the entirety of the variability observed in health benefits of these compounds^59^. Our data illustrate that there might be other factors at play: for example, there is evidence indicating that individuals with high cardiorespiratory fitness tend to have higher CO_2_ cerebrovascular reactivity^60,61^. While we did not assess the fitness of the participants in the present study, one possible explanation for the differential responses observed between sub-groups was that flavanols may have improved the cerebrovascular reactivity of less fit individuals to levels similar to those of potentially highly fit individuals. Future work should formally address this hypothesis, which may have significant translational implications.

## Conclusions

We have demonstrated that acute flavanol intake can improve efficiency in blood oxygenation (amplitude and speed) during hypercapnia in frontal cortical areas of young healthy subjects and that it is likely to contribute to improvements in cognitive function, but only when cognitive demands are high. We also show that only individuals with lower baseline cerebrovascular reactivity benefit from flavanol intake, with acute improvements in cerebral vascular function and cognitive performance. We suggest that the underlying mechanisms at play centrally may be similar to the ones detected in the peripheral vasculature, through hypercapnia-induced increases in NO release from the endothelium in cerebral arteries. Future work should confirm this by conducting studies in animal models where direct assessments of NO levels in the cerebral vasculature are possible in real time during hypercapnic challenges.

The findings reported here can have important future implications for using dietary strategies containing plant-derived flavanols for enhancement of blood oxygenation and cognitive performance in healthy populations, as well as for populations at higher risk (e.g., smokers; hypertensives; diabetics; older adults) or to help recover and treat brain injuries and disease. Most importantly, our data can potentially open new avenues for precision-medicine research with regard to understanding individual responses to flavanol intake and helping to identify populations that might benefit the most from these interventions.

### Limitations

fNIRS measures have high temporal sampling, which allow for studying the dynamics of oxygenation effects, but only provide information about superficial areas of the brain, which is a common limitation to all diffuse optical imaging measures. We also only focused on frontal cortical areas and observed that, within this region, the effects of flavanols in response to hypercapnia seemed to be relatively homogeneous. However, we are unable to establish whether there are regional differences across the brain. Whilst previous studies suggest that flavanols can increase NO in humans in an acute manner, in the present study we have not assessed NO species and cannot conclude that flavanol-induced responsiveness to hypercapnia is specifically linked to this mechanism. Other important limitations of this study are the low number of participants, particularly to address cognitive outcomes, and the exclusion of females. Additionally, the evaluation of efficacy of flavanol intake in an at-risk population (e.g. older adults) would have more ecological validity and this should be addressed in future work.

## Methods

### Participants

Eighteen healthy male volunteers (18–45 years old) were recruited from the University of Birmingham and surrounding areas according to the following inclusion criteria: (i) non-smokers; (ii) normotensive; (iii) no history of cerebrovascular, cardiovascular or respiratory disease; (iv) no allergies or intolerances to ingredients present in cocoa powders; (v) not taking long-term medication (e.g., hyperlipidaemia or on antibiotics for the previous 3 months); (vi) not suffering from blood-clotting disorders; (vii) no known infections at the time of the study; (viii) not on a weight-reducing regimen (Table 1). Females were excluded from the study to ensure a more homogenous sample and to minimize the impact of hormonal fluctuations during the menstrual cycle on vascular outcomes. Volunteers were asked to refrain from the following for the 24 h before the study: (1) consumption of polyphenol-rich foods including fruits, vegetables, cocoa, chocolate, coffee, tea, fruit juices and wine; (2) consumption of nitrates, including foods such as beetroot, spinach, lettuce, rocket, celery, parsley and cabbage (defined as containing > 50 mg nitrates/100 g fresh weight); (3) performing any type of vigorous physical exercise; and (4) consuming any alcoholic beverage. Volunteers were further asked to fast for 12 h before each study visit. All research was performed in accordance with relevant guidelines and regulations. Informed consent was obtained from all the participants. The study was approved by the University of Birmingham Science, Technology, Engineering and Mathematics Ethical Review Committee (ERN_17-1591).

**Table 1:** Participant baseline characteristics (N = 18).

	Mean	SD
Age (years)	23.9	7.3
Weight (kg)	71.9	10.2
BMI (kg/m^2^)	22.7	2.0
Systolic blood pressure (mmHg)	117.9	9.7
Diastolic blood pressure (mmHg)	61.7	6.7

### Study design

The study was based on an acute, randomised, placebo-controlled, double-blind, cross-over design. After ascertaining eligibility, participants attended two visits, separated by a minimum of two weeks, in which they consumed either a high-flavanol cocoa drink (HF) or a low-flavanol (LF) cocoa (control) drink, to which they, as well as the experimenters, remained blinded throughout the study (for composition of the two cocoa interventions see Table 2). In a third visit participants visited the University of Birmingham Imaging Centre to have a structural MRI brain scan. Participants were assigned a code, corresponding to the flavanol intervention for each visit according to a random allocation sequence. Compliance to the food and fasting requirements were assessed using 24-h dietary recall conducted at the beginning of each visit. On each visit, volunteers rested for 20 min in supine position in a quiet, temperature-controlled room before baseline measures were taken in this order: (i) diastolic and systolic blood pressure (BP); (ii) brachial artery FMD; (iii) fNIRS of frontal cortex oxygenation/deoxygenation at rest and during hypercapnia (5% CO_2_). Following baseline measurements volunteers consumed either the high-flavanol intervention or control low-flavanol intervention (within 10 min). Two hours post intake, BP, FMD and fNIRS-based optical reactivity to the hypercapnic challenge were assessed, as well as cognitive function using a Modified Stroop Task^34^. Cognitive function was assessed only at the 2-h point to minimize practice effects. During the time between ingestion of the beverage and post measurements, optical sources and detector locations were digitized (for each visit) using a Polhemus FASTRAK 3D Digitizer (Polhemus, Colchester, VT). The design of the cognitive tasks was not compatible with accurate fNIRS analysis, mainly due to the variable duration of the task between subjects and the small number of trials. Methods and results regarding the standard FMD and BP measures are included in supplementary materials.

**Table 2:** Composition of cocoa interventions (8.3 g per individual dose) containing high and low flavanol content used in the acute study.

		High-flavanol	Low-flavanol
Total polyphenols	mg	1052.5	143.4
Total flavanols	mg	681.4	4.1
Procyanidins (dimers-decamers)	mg	495.9	ND
(−)-Epicatechin	mg	150.0	< 4
(−) and (+)-Catechin	mg	35.5	< 4
Theobromine	mg	179.8	179.8
Caffeine	mg	19.5	19.3
Fat	g	1.2	0.9
Carbohydrates	g	4.6	4.4
Protein	g	1.9	1.9
Fibre	g	1.3	2.9
Energy	kcal	19.1	17.0

### Flavanol-containing interventions

Intervention beverages were prepared immediately prior to consumption by dissolving 8.3 g of cocoa powders in 300 ml of room temperature bottled water containing low levels of nitroso species (Buxton). The cocoa powders used are commercially available (Barry Callebaut, Switzerland): the low-flavanol control is a fat-reduced cocoa powder alkalized (commercial name: 10/12 DDP Royal Dutch) and the high-flavanol cocoa powder a non-alkalized fat reduced powder (‘Natural Acticoa’). Both are matched for macronutrient and micronutrient content (including caffeine and theobromine). The high-flavanol cocoa delivered 150 mg of (−)-epicatechin and 35.5 mg of catechin (flavanol monomers), whilst the low-flavanol intervention delivered < 4 mg of both monomers (Table 2). The dose of flavanol monomers used in the present study is in line with previous studies showing acute efficacy in modulating human endothelial function^6,8,39^ and plasma NO levels^40^. A similar dose of (−)-epicatechin could be achieved through diet by consuming foods rich in flavanols, particularly apples, black grapes, blackberries, cherries, raspberries, pears, pulses, green tea and red wine^62^. Total levels of polyphenols in the powders were assessed by a Folin-Ciocalteu reagent calorimetric assay as described previously^63^. Individual monomer levels and procyanidin levels as well as levels of methylxanthines, were confirmed by HPLC as described previously^64,65^. Maximal levels of flavanol monomers metabolites are reached in the human circulation at approximately 2 h post-intake^13^. Individual doses of cocoa powder were kept at − 20 °C and identified by a three-digit code to ensure double-blindness. Intervention beverages were identical in texture, consistency and taste, and were delivered to participants in an opaque container with an opaque straw. Participants and researchers involved in data collection and analysis were blind to the intervention conditions until all data analysis was completed.

### Optical recording

Optical data were recorded using an Imagent2 fNIRS device (ISS Inc., Champaign, IL.), based on 32 sources (16 0.4 mm optical fibres connected to laser-diodes emitting light at 690 nm and 16 at 830 nm) and 14 detectors (3 mm fibre-optic bundles linked to photomultiplier tubes, PMTs). Source and detector fibres were held in place using custom-built helmets matched to a range of head circumferences. Hair was combed away from the points of contact between detectors and scalp, whilst the smaller source fibres allowed them to be placed directly through the hair onto the scalp. Source fibres were paired such that each location was illuminated by two fibres connected to one 830 nm and one 690 nm laser, but the two fibres were never on at the same time. Each detector received light from 16 time-multiplexed sources, which stayed on for a period of 1.398 ms and off for 20.971 ms, yielding an effective sampling rate of 44.704 Hz. To avoid crosstalk between channels (i.e., source-detector pairs), the optical montage ensured that a detector could not receive light from more than one source contacting the head at less than 70 mm distance at any one time.

### Structural MRI acquisition and co-registration with optical data

All participants underwent a high-resolution structural MRI scan in a 3-T Philips Achieva MRI scanner (Philips Medical Systems, Best, Netherlands). A whole-head T1-weighted anatomical image was produced using an MPRAGE sequence with 1 mm^3^ resolution. All MRI scanning was carried out either for the purpose of this study or acquired from previous studies with the written consent of the participant and original researcher, with the following parameters: flip angle = 8◦, TE = 3.8 ms, TR = 8.39 ms, inversion time = 1150 ms, 175 sagittal slices, voxel size 1.0 × 1.0 × 1.0 mm with a field of view of 288 × 232 × 175 mm (FH x AP x RL). The structural MRI was used for co-registration of optical data onto each individual’s anatomy^66^ using nasion and pre-auricular points as references, the spatial locations of sources and detectors were digitized. Digitization points were then co-registered with T1 structural MRI scans, following procedures described by^66^. This approach has been shown to have errors of less than 4 mm^66,67^. See supplementary Figure S3 for an optical montage superimposed on the structural MRI of a representative participant.

### Hypercapnia

Hypercapnia was induced with 5% CO_2_ (in air) via the open-circuit Douglas bag method. Once participants were instrumented with the fNIRS device, baseline data were collected while breathing room air for 4 min, followed by 4 min breathing the 5% CO_2_ gas mixture (hypercapnia), then a 2-min recovery period breathing room air^68^. Respiratory gases were analysed for changes in end-tidal CO_2_ using a continuous gas analyser (ML206, AD Instruments, Dunedin, New Zealand). Throughout this hypercapnic cerebrovascular reactivity procedure, participants remained seated whilst optical data were recorded.

### Cognitive performance

Cognitive performance was assessed using a modified Stroop task^34,69^ (see supplementary Fig. S4). The task assesses cognitive processes related to selective attention and prepotent response inhibition during decision making, with increasing task demands across levels. It involves the presentation of colour words (e.g., RED) displayed in either congruent (e.g., red) or incongruent (e.g., blue) colours, alongside two control trial blocks in which only one dimension of the stimuli is presented: either colour words presented in black or colour blocks without words (Fig. S4A,B and C,D). At the most difficult level (Fig. S4 panels G,H), the modified Stroop presents task blocks that include two forms of conflict (at stimulus classification and at response selection, (double Stroop, Fig. S4G, H), rather than one form of conflict (at stimulus classification) as used in the standard Stroop Task (Fig. S4E,F). This allowed for a comparison between simpler and more demanding conditions. Previous work demonstrated that performance in the double-Stroop task results in lower performance (i.e., longer response times and lower accuracy) than the standard Stroop task, but it can be improved by interventions (e.g., exercise) in young healthy adults^34^, confirming the suitability of this task for the current study. The tasks involved four blocks of 40 trials in which participants responded by pressing the bottom left (‘z’) or right (‘/’) key, corresponding to response options displayed on the screen (supplementary Fig. S4). Note that the simplest two task blocks tested identification of a colour word presented in a neutral colour (word task) and recognition of the colour of a patch (colour task). Blocks 3 and 4 both required participants to identify the colour of the text whilst ignoring or inhibiting the prepotent response to the word name. The third block (Stroop task) had response options printed in a neutral colour (black), whilst the fourth block added a further form of conflict by presenting the response options in conflicting coloured text, requiring participants to inhibit a second prepotent response stimulus (“double-Stroop”).

### Data analysis

#### Optical Processing and hemodynamic responses during hypercapnia

Optical data were obtained for 10 min epochs starting 4 min before the beginning of the 5% CO_2_-breathing challenge. Channel pairs (composed of a 690 and 830 nm pair of channels from the same source and detector locations) with an average AC count < 100 digitizing units for either wavelength were discarded. For the remaining channels, raw fNIRS signals were normalised by dividing each value by the mean value across the time points for each block and channel. To determine the overall haemodynamic response across CO_2_ manipulations, data were corrected for vascular pulsation^70,71^. Pulse-corrected data were then motion corrected^72^ and low-pass (zero-phase shift) filtered at 0.05 Hz. The data were then down-sampled to one value every 9 s, and the changes in light intensities for each sample and channel pair were transformed into oxy- and deoxy-haemoglobin concentration changes using the Beer–Lambert law^73^. Finally, to eliminate the effects of non-brain phenomena, the time course of the response for channels with the shortest source-detector distance (< 15 mm, unlikely to be affected by brain phenomena) was regressed out from each channel data^74^. In-house software Opt-3D^75^ was used to merge channels whose diffusion paths intersected within a given brain volume (modelled as a curved ellipsoid path^76^). Only source-detector distances within a 20–50 mm range were used for analysis, in order to focus on signal extraction from deeper brain regions and exclude superficial tissue effects, dominant at shorter source-detector distances. An 8-mm spatial filter was applied to spatially reconstructed source-detector data, which were projected onto the axial surface of a model brain image in Talairach space. The data from 1 volunteer were excluded from fNIRS analysis due to excessive noise.

#### Statistics/power analysis

fNIRS and FMD and cognitive data were analysed using two-way repeated measures ANOVA with intervention (low- or high-flavanol) and time (0, 2 h) as within-subject factors. Cognitive data were analysed using a two-way repeated measures ANOVA with intervention (low- or high-flavanol) and task levels (Word, Colour, Stroop, Double Stroop) as within-subject factors. fNIRS data were averaged across the whole cortical area recorded for each participant. Planned a priori comparisons between high and low-flavanol were tested by 2-tailed t-tests. Significance was defined as *p* < 0.05 (95% CI) for all outcome measures. Sample size was estimated based on previous data from our laboratory on flavanol acute changes in brachial FMD in this population (mean = 5.47; SD = 1.34): the selected sample size (N = 18) was expected to afford us a 95% probability of detecting a difference (i.e., power = 0.95) of 0.6% FMD (the expected effect size) due to flavanol intake, using an alpha value of α = 0.05.

## Supplementary information

Supplementary Figures.

## Data Availability

The datasets generated during and/or analysed during the current study are available from the corresponding author on reasonable request.
