# Supplementary material for: Dietary flavanols improve cerebral cortical oxygenation and cognition in healthy adults
Source: Sci Rep. 2020 Nov 24;10:19409. doi: 10.1038/s41598-020-76160-9 (PMC7687895; doi:10.1038/s41598-020-76160-9)
Supplement: Supplementary file 1 — Supplementary Figures. [file 41598_2020_76160_MOESM1_ESM.docx]

**Dietary flavanols improve cerebral cortical oxygenation and cognition in healthy adults**

Gabriele Gratton^1,2^, Samuel R. Weaver^3^, Claire V. Burley^3^, Kathy A. Low^1^, Edward L. Maclin^1^, Paul W. Johns^4^, Quang S. Pham^4^, Samuel J.E. Lucas^3,5^, Monica Fabiani^1,2^, Catarina Rendeiro^3,5^*

^1^Beckman Institute for Advanced Science and Technology, University of Illinois at Urbana-Champaign, Urbana, Illinois, USA

^2^Department of Psychology, University of Illinois at Urbana-Champaign, Urbana, Illinois, USA

^3^School of Sport, Exercise and Rehabilitation Sciences, University of Birmingham, Birmingham B15 2TT, UK

^4^Abbott-Nutrition Division, Research and Development, 3300 Stelzer Road, Columbus, OH 43219, USA

^5^Centre for Human Brain Health, University of Birmingham, Birmingham, UK

**Supplementary Information**

**Methods**

*Brachial Flow-mediated dilation*

Although this study focused on the effects of cocoa flavanols on brain vasoreactivity and cognition, we also investigated whether they affected peripheral circulation, to make sure that previous effects on peripheral endothelial function were replicated^1,2^.  Flow-mediated dilatation (FMD) of the brachial artery was used to assess peripheral endothelial-dependent vasodilatory responses prior to and following the flavanol interventions. FMD measurements were taken following standard guidelines^3,4^ using a 15-4Mhz (15L4 Smart MarKᵀᴹ) transducer attached to a Terason Duplex Doppler system (Usmart 3300 NexGen Ultrasound; Terason, United States) in combination with an automatic edge-detection and wall-tracking software (Cardio-vascular Suite, Quipu), which allows for continuous measurement of diameter and blood velocity throughout the FMD assessment. Participants were asked to rest for 20 min in the supine position in a quiet, temperature-controlled room, following which the brachial artery was imaged longitudinally at 5-10 cm proximal to the antecubital fossa. After baselines images were captured for 60 s, a blood pressure cuff was placed around the forearm and was inflated to 220 mmHg. After 5 min of forearm occlusion, the cuff pressure was rapidly released to allow reactive hyperaemia to occur, with continuous image collection for 5 min post-pressure release. A researcher, blinded to condition allocation and measurement details, analysed all image files. Peak diameter was defined as the largest diameter recorded after occlusion was released. FMD response was calculated as the relative diastolic diameter change between baseline and peak diameter. FMD responses for 16/18 participants were analysed, because the ultrasound images for 2 of the volunteers were not captured during the peak dilation due to movement.

FMD was analysed using two-way repeated measures ANOVA within subjects with time (0, 2 h) and intervention (low- or high-flavanol) as main factors. Post hoc comparisons were carried out using Bonferroni correction for multiple comparisons. Signiﬁcance was deﬁned as *p*<0·05 (95% CI) for all outcome measures

**Results**

The FMD results are presented in Supplementary Figure S2. Consistent with previous work^1^ we show that high-flavanol intake was associated with an increase in FMD relative to low-flavanols administration.  This was confirmed by a two-way repeated measure ANOVA showing a significant interaction, *F*(1, 15)=10.28, *p*=.005.  Planned contrasts indicated a significant increase (~1% FMD) from 0 to 2 hours for the high-flavanol intervention (*p*=.033). At 2 hours, a significant difference between the high- and low-flavanol interventions was detected (*p*<0.001), but no differences at baseline (0 h) (*p*=.52).

Across individuals, the peripheral vascular effects of flavanols were moderately, and non-significantly, associated (*r*=.433) with the cerebrovascular effects (this correlation was based on the interaction terms measured in each individual in the FMD and oxy-haemoglobin concentration changes as a function of CO_2_-breathing from before to after high- vs. low-flavanols administration).

**Figures**

**
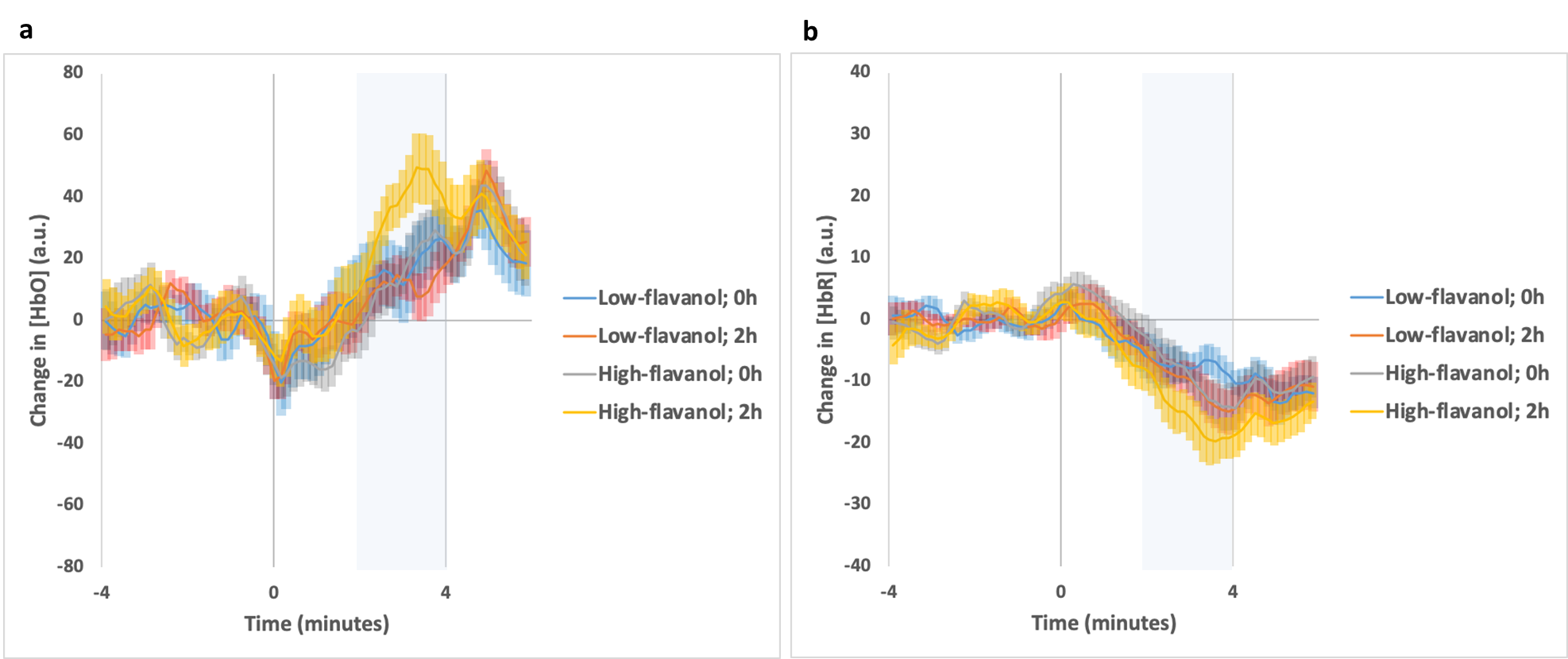
**

**Figure S1**: **Haemodynamic responses in frontal cortical regions during hypercapnia (5% CO_2_) both before (0 h) and after (2 h) intake of either a low- or high-flavanol dietary intervention**. Time course for Oxygenated (a) and Deoxygenated-haemoglobin (b) are presented as averages across participants (N=17) and across frontal brain locations (Mean ± SEM).


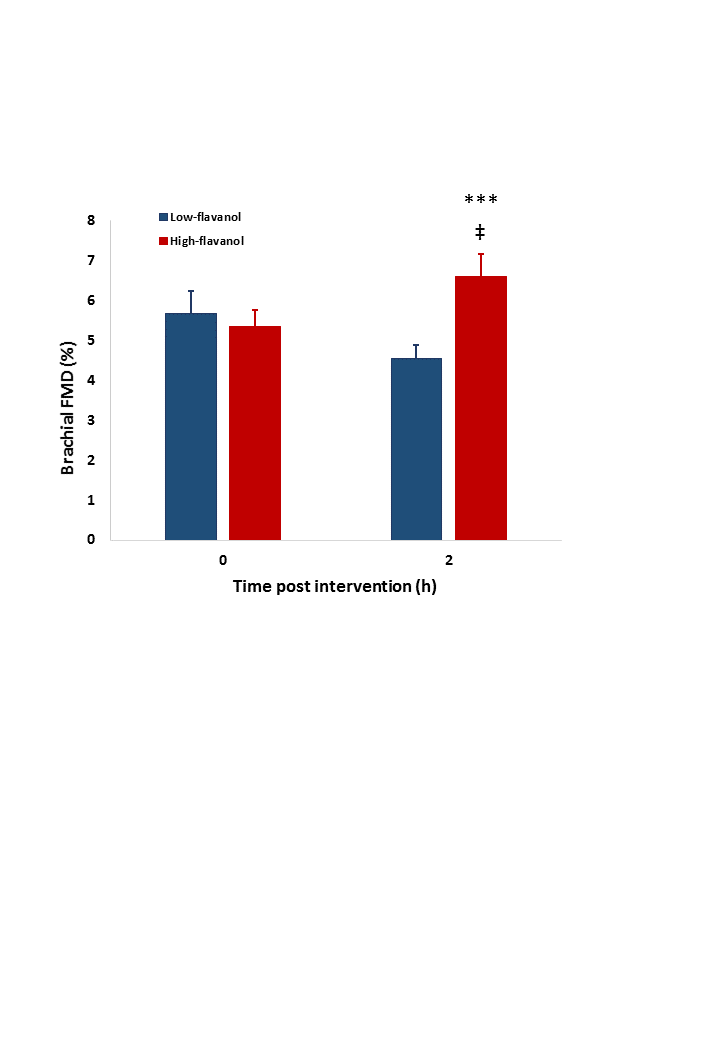


**Figure S2: Flow-mediated dilatation (FMD) of the brachial artery both before (0 h) and after (2 h) intake of either a low- or high-flavanol dietary intervention.** A 2-way ANOVA revealed a significant interaction between flavanol intervention and time [*F*_1, 15_=10.28, *p*=.005], with a significant increase of ~1% FMD from 0 to 2 hours only observed for the high-flavanol intervention (ǂ *p*=.033). At 2 h (but not at 0 h) there was also a significant difference between high and low-flavanol interventions (*** *p*<.001). The data is presented as Mean ± SEM.

**
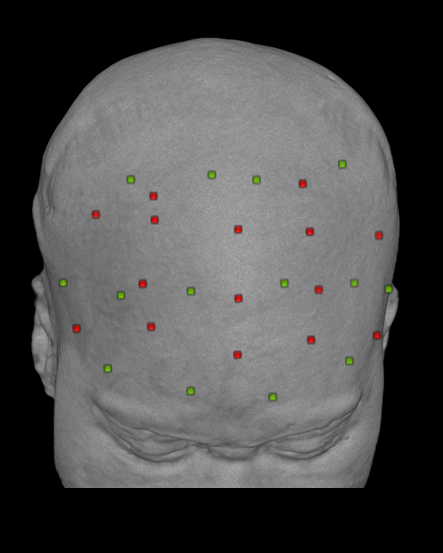
**

**Figure S3: Optical Montage targeting frontal cortical regions.** Distribution of the sources (in red) and detectors (in green) used for data collection over the MR-rendered scalp of a representative participant.


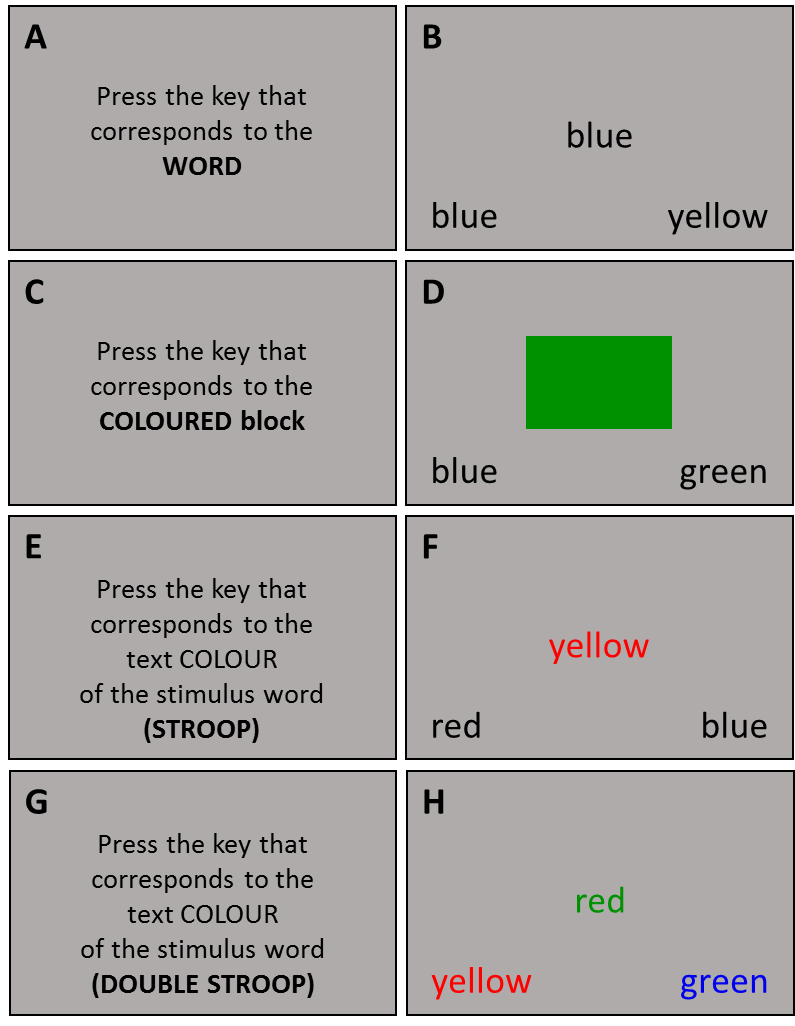


**Figure S4:**  **Design of a modified Stroop Task, as previously described^5^.** This includes blocks of increasing difficulty. The simplest two task blocks present no conflict, requiring the identification of a colour word presented in a neutral (black) colour (**Word task, A-B**) and recognition of the colour of a patch (**Colour** **task**, **C-D**). Blocks 3 and 4, with progressively increased cognitive demand, require participants to identify the colour of the text whilst ignoring or inhibiting the prepotent response to the word name. Block 3 has response options printed in a neutral (black) colour (**Stroop task, E-F**), presenting only one form of conflict at stimulus classification. Block 4 added a further level of conflict by presenting response options in conflicting coloured text requiring participants to inhibit a second prepotent response stimulus (**Double-Stroop task, G-H**).

**References**

1 Sansone, R. *et al.* Cocoa flavanol intake improves endothelial function and Framingham Risk Score in healthy men and women: a randomised, controlled, double-masked trial: the Flaviola Health Study. *Br J Nutr* **114**, 1246-1255, doi:10.1017/S0007114515002822 (2015).

2 Schroeter, H. *et al.* (-)-Epicatechin mediates beneficial effects of flavanol-rich cocoa on vascular function in humans. *Proc Natl Acad Sci U S A* **103**, 1024-1029, doi:10.1073/pnas.0510168103 (2006).

3 Corretti, M. C. *et al.* Guidelines for the ultrasound assessment of endothelial-dependent flow-mediated vasodilation of the brachial artery: a report of the International Brachial Artery Reactivity Task Force. *J Am Coll Cardiol* **39**, 257-265, doi:10.1016/s0735-1097(01)01746-6 (2002).

4 Thijssen, D. H. *et al.* Assessment of flow-mediated dilation in humans: a methodological and physiological guideline. *Am J Physiol Heart Circ Physiol* **300**, H2-12, doi:10.1152/ajpheart.00471.2010 (2011).

5 Lucas, S. J. *et al.* Effect of age on exercise-induced alterations in cognitive executive function: relationship to cerebral perfusion. *Exp Gerontol* **47**, 541-551, doi:10.1016/j.exger.2011.12.002 (2012).
